# Humanistic care and psychological counseling on psychological disorders in medical students after COVID-19 outbreak: A protocol of systematic review

**DOI:** 10.1097/MD.0000000000021484

**Published:** 2020-08-14

**Authors:** Hao Tian, Yu Xue, Rong-rong Yao, Yu Yan, Yong Xue, Da-yin Chen, Fan-bo Wang, Chun-feng Li, Qing-hui Ji

**Affiliations:** a Department of General Surgery, First Affiliated Hospital of Jiamusi University; b Department of Obstetrics and Gynecology, Second Affiliated Hospital of Jiamusi University; c Department of Interventional Radiology; d Department of Gastroenterology, First Affiliated Hospital of Jiamusi University; e School of Life Sciences, Jiamusi University; f Department of Urology; g Department of Videography; h First Ward of Orthopedics Department, First Affiliated Hospital of Jiamusi University, Jiamusi, China.

**Keywords:** COVID-19, humanistic care, psychological counseling, psychological disorders

## Abstract

**Background:**

The objective of this study is to investigate the effects of humanistic care and psychological counseling (HCPC) on psychological disorders (PD) in medical students after coronavirus disease 2019 (COVID-19) outbreak.

**Methods:**

We will search randomized controlled trials or case-controlled studies of HCPC on PD in medical students after COVID-19 outbreak in the following electronic databases: PUBMED/MEDLINE, EMBASE, Cochrane Library, CINAHL, AMED, WANGFANG, and CNKI. The time is restricted from the construction of each database to the present. All process of study selection, data collection, and study quality evaluation will be carried out by two independent authors. Any different opinions will be solved by a third author through discussion. We will employ RevMan 5.3 software to conduct statistical analysis.

**Results:**

This study will provide a better understanding of HCPC on PD in medical students after COVID-19 outbreak.

**Conclusions:**

This study may offer strong evidence for clinical practice to treat PD in medical students after COVID-19 outbreak.

**Study registration:**

CRD42020193199.

## 1 Introduction

Coronavirus disease 2019 (COVID-19) is a new coronavirus that has never been found in humans before.^[1–3]^ It can cause severe acute respiratory syndrome and spread rapidly worldwide.^[4–8]^ It manifests as fever or chills, cough, shortness of breath, fatigue, muscle or body aches, sore throat, congestion or runny nose, and dyspnea.^[9–11]^ If it cannot be treated fairly well, it can further develop to pneumonia, severe acute respiratory syndrome, kidney failure, and even death.^[12–15]^ According to the report of World Health Organization, by June 24, there are more than 9.1 million cases of COVID-19 in total, and more than 470,000 deaths worldwide.^[16]^

Outbreak of COVID-19 is associated with considerable psychological disorders (PD), such as depression, anxiety, and fear in general public, specific communities, or medical students, especially when the infection rate and deaths are considerable.^[17–23]^ After the period of COVID-19 outbreak, tackling PD of medical students is crucial. Studies suggested that humanistic care and psychological counseling (HCPC) can effectively treat PD in medical students after COVID-19 outbreak.^[24–29]^ However, no systematic review has investigated this issue. Thus, this systematic review protocol will appraise the effects of HCPC on PD in medical students after COVID-19 outbreak.

## 2 Methods

### 2.1 Study registration

We registered this study protocol on CRD42020193199. We reported it based on the guideline of Preferred Reporting Items for Systematic Reviews and Meta-Analyses Protocols.^[30]^

### 2.2 Inclusion and exclusion criteria for study selection

#### 2.2.1 Types of studies

We will include randomized controlled trials (RCTs) or case-controlled studies (CCSs) of HCPC on PD in medical students after COVID-19 outbreak. We will not consider other studies, such as animal study, review, editorial comment, case report, and case series.

#### 2.2.2 Types of participants

The target population is medical students with confirmed PD after COVID-19 outbreak irrespective of ethnicity, sex, and severity of PD.

#### 2.2.3 Types of interventions

The experimental interventions are any forms of HCPC combination.

Besides any types of HCPC, no limitations will be made on comparators.

#### 2.2.4 Types of outcome measurements

The primary outcomes are depression and anxiety, as reported by any scales in the trial, such as Hamilton Depression Rating Scale and Hamilton Anxiety Rating Scale.

The secondary outcomes are mental disorder, panic disorder, health-related quality of life, and adverse events.

### 2.3 Search methods for study selection

#### 2.3.1 Electronic databases

We will search the following electronic databases from the construction of each database to the present: PUBMED/MEDLINE, EMBASE, Cochrane Library, CINAHL, AMED, WANGFANG, and CNKI. We will build search strategy for Cochrane Library and will be presented in Table 1. Similar search strategy for other electronic databases will be adapted. As for Chinese databases, equivalent search words will be applied.

**Table 1:**
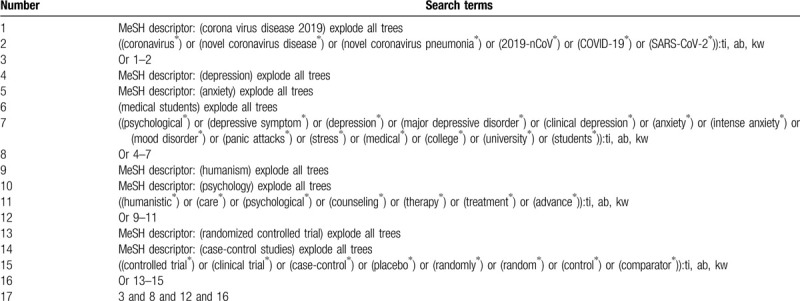
Details of search strategy of Cochrane Library.

#### 2.3.2 Other resources

We will also check other resources, such as conference abstracts, dissertation/thesis, and reference lists of relevant review.

### 2.4 Study selection and data extraction

#### 2.4.1 Study selection

Two authors will independently carry out study selection. Any disagreements will be solved by a third author through consultation. All searched records will be imported to the citation management software (EndNote 7.0), and all duplications will be eliminated. Then, titles and abstracts of all potential studies will be scanned to remove unrelated studies. After that, we will carefully check full-text of all potential articles according to the eligibility criteria. We will present the process of study selection in a flowchart.

#### 2.4.2 Data extraction and management

Two authors will independently undertake data extraction using a standard data extraction sheet. Any confusion will be resolved by a third author through discussion. The extracted information includes general information (such as title, first author, and time of publication), study information (such as design, setting, methods, and sample size), patient information (such as ethnicity, age, sex, diagnostic criteria, and eligibility criteria), outcomes (such as primary and secondary outcomes, and follow-up data), and other information (such as funding and conflict of interest). If there is unclear or missing data, we will contact primary authors to obtain it by email or telephone or fax.

#### 2.4.3 Risk of bias assessment

Two independent authors will carry out study quality assessment using Cochrane Risk of Bias Tool for RCTs, and Newcastle-Ottawa Quality Assessment for CCSs, respectively. Any discrepancies will be settled down by a third author through discussion or consultation.

### 2.5 Statistical analysis

#### 2.5.1 Data synthesis

This study will utilize RevMan 5.3 software to perform statistical analysis. We will estimate treatment effect of dichotomous outcomes as risk ratio and 95% confidence intervals (CIs), and that of continuous outcomes as mean difference (MD) or standardized MD and 95% CIs. We will identify statistical heterogeneity using *I*^2^ test. *I*^2^ ≤50% means reasonable heterogeneity, and we will employ a fixed-effect model. If sufficient data are extracted from eligible trials, we will conduct meta-analysis based on similarity in study information, patient information, interventions, controls, and outcomes. *I*^2^ > 50% exerts considerable heterogeneity, and we will place a random-effect model. In addition, we will carry out a subgroup analysis to explore its sources. If the meta-analysis is not possible, we will perform a narrative synthesis of study findings.

#### 2.5.2 Unit of analysis

We will only collect and analyze data from the first period of eligible study if cross-over study is included.

#### 2.5.3 Subgroup analysis

We will preside over a subgroup analysis in accordance with different study information, subject characteristics, specifics of interventions and controls, and study quality.

#### 2.5.4 Sensitivity analysis

We will undertake a sensitivity analysis to check the robustness of study findings by removing low quality studies.

#### 2.5.5 Reporting bias

When more than 10 eligible studies on the same outcome are included, we will investigate reporting bias using Funnel plot and Egger's linear regression test.^[31,32]^

#### 2.5.6 Overall quality of evidence

Two authors will independently appraise the overall quality of evidence for each outcome using the Grading of Recommendations Assessment, Development and Evaluation approach.^[33]^ It will be further divided into five aspects of high, moderate, low or very low quality.^[33]^

### 2.6 Dissemination and ethics

We expect to publish this study on a peer-reviewed journal or a conference meeting. This study will not require ethic approval, because it will not collect individual patient data and privacy.

## 3 Discussion

The outbreak of COVID-19 pandemic creates challenges for clinicians and scientific researchers.^[1,2]^ It also brings tremendous PD for general population, doctors, nurses and medical students.^[17–23]^ Previous studies reported that HCPC can benefit PD in medical students after COVID-19 outbreak.^[24–29]^ However, there is no systematic review focusing on this topic, and evidence-based medicine literature is very necessary to explore its effects. Thus, in this study, we will summarize recent associated studies to appraise the effects of HCPC on PD in medical students after COVID-19 outbreak. The findings of this study may yield helpful evidence for both clinicians and health-related policy makers.

## Author contributions

**Conceptualization:** Hao Tian, Yu Xue, Rong-rong Yao, Da-yin Chen, Fan-bo Wang, Qing-hui Ji.

**Data curation:** Hao Tian, Rong-rong Yao, Yu Yan, Yong Xue, Da-yin Chen, Fan-bo Wang, Chun-feng Li.

**Formal analysis:** Hao Tian, Yu Xue, Yong Xue, Chun-feng Li.

**Investigation:** Qing-hui Ji.

**Methodology:** Hao Tian, Rong-rong Yao, Yong Xue, Da-yin Chen, Fan-bo Wang.

**Project administration:** Qing-hui Ji.

**Resources:** Hao Tian, Yu Xue, Rong-rong Yao, Yu Yan, Yong Xue, Da-yin Chen, Fan-bo Wang, Chun-feng Li.

**Software:** Hao Tian, Yu Xue, Rong-rong Yao, Yu Yan, Yong Xue, Da-yin Chen, Fan-bo Wang, Chun-feng Li.

**Supervision:** Qing-hui Ji.

**Validation:** Hao Tian, Yu Xue, Yu Yan, Yong Xue, Da-yin Chen, Fan-bo Wang, Chun-feng Li, Qing-hui Ji.

**Visualization:** Hao Tian, Rong-rong Yao, Yu Yan, Yong Xue, Qing-hui Ji.

**Writing – original draft:** Hao Tian, Yu Xue, Rong-rong Yao, Yu Yan, Yong Xue, Da-yin Chen, Fan-bo Wang, Chun-feng Li, Qing-hui Ji.

**Writing – review & editing:** Hao Tian, Yu Xue, Yong Xue, Fan-bo Wang, Chun-feng Li, Qing-hui Ji.
